# Multilevel Engagements of Pharmacists During the COVID-19 Pandemic: The Way Forward

**DOI:** 10.3389/fpubh.2020.561924

**Published:** 2020-12-08

**Authors:** Tauqeer Hussain Mallhi, Aroosa Liaqat, Arooj Abid, Yusra Habib Khan, Nasser Hadal Alotaibi, Abdulaziz Ibrahim Alzarea, Nida Tanveer, Tahir Mehmood Khan

**Affiliations:** ^1^Department of Clinical Pharmacy, College of Pharmacy, Jouf University, Sakaka, Saudi Arabia; ^2^Punjab University College of Pharmacy, University of the Punjab, Lahore, Pakistan; ^3^Primary and Secondary Healthcare Department, Tehsil Headquarter Hospital, Rawalpindi, Pakistan; ^4^Tehsil Headquarter Hospital, Jaranwala, Faisalabad, Pakistan; ^5^Institute of Pharmaceutical Sciences, University of Veterinary and Animal Sciences, Lahore, Pakistan

**Keywords:** COVID-19, coronavirus, pharmacist, clinical pharmacist, community pharmacist, industrial pharmacist, pharmacy services, pandemic

## Abstract

Severe acute respiratory syndrome caused by the novel coronavirus (SARS-CoV-2) was first reported in China in December 2019 which was later declared to be a public health emergency of international concern by the World Health Organization (WHO). This virus proved to be very contagious resulting in life-threatening respiratory intricacies posing overall public health and governance challenges. Amid the coronavirus pandemic and the unprecedented increase in healthcare demands, only inventive and adaptive practice among healthcare professionals is the need of the hour. Pharmacy services are an important mainstay in the public health and have considerable potential to combat the coronavirus disease 2019 (COVID-19) pandemic. Pharmacists working in several localities and health facilities are linked to patients either directly or indirectly. They can act swiftly in public health response such as drafting professional service guidance to pharmacists working in various healthcare facilities, ensuring effective medicine supply system, monitoring and resolving drug shortage issues, establishing and promoting remote pharmacy services, counseling the public on infection prevention basics, educating about proper use of personal protective equipment, discouraging self-medication, participating in clinical trials, small-scale manufacturing of sanitizers and disinfectants, busting the prevailing myths, and conducting drug evaluation and active surveillance. These interventions will help ease unprecedented burden on healthcare facilities during the ongoing pandemic and eventually will add value to patients and the healthcare system. The current manuscript accentuates the potential roles and activities that pharmacists can initiate in various healthcare facilities to help in relieving pressure on the overwhelmed healthcare system. The information and suggestions offered in this review could help in the restructuring of existing pharmacy services by governments, public health bodies, and policy makers in response to the COVID-19 pandemic. Moreover, this manuscript will underscore any unrealized potential among pharmacists working in various sectors including community, hospital, industry, and drug regulatory authorities.

## Background

Pharmacists are one of the most trusted professions worldwide alongside firefighters, nurses, teachers, and doctors (1, 2). During the current pandemic when the healthcare system is collapsing amid the unprecedented number of coronavirus disease 2019 (COVID-19) cases, pharmacists can play a pivotal role in disease prevention, management, and containment (3). They work in several localities and are linked to the patients, either directly or indirectly (4). Health authorities across various countries are recognizing the value of community pharmacists in the healthcare system due to their availability and accessibility to the public. However, pharmacy services in infectious disease control and pandemic are least appreciated.

During the ongoing COVID-19 crisis where clinicians and nurses are overburdened, pharmacists are well-situated to offer collaborative and complementary expertise alongside current models of care. However, the capabilities of pharmacists are under-recognized both by patients and physicians. Practitioners report strong mutual respect for pharmacists as allied health professionals, but communication between them could be strengthened (2). Moreover, there is a dearth of investigations to ascertain the impact of pharmacy services in controlling infectious diseases. There is a dire need to potentially utilize the services of pharmacists working in community, hospital, industry, and drug regulatory authorities. In addition, integrated efforts of pharmacists working in various settings with clinicians, nurses, and public health officials will strengthen the ongoing maneuvers to contain COVID-19. The purpose of this manuscript is to underscore the pivotal contributions of pharmacists which could potentially assist to curb the growing encumbrance of the disease.

Since community pharmacies, hospitals, pharmaceutical industries, and drug regulatory authorities are exempted from the current lockdown which is being observed by most of the countries, effective utilization of pharmacist's services will be of paramount importance to tackle the quadruple burden associated with the pandemic. A pharmacist, being a most trusted and accessible healthcare professional, can be utilized to combat the chaos attributed to the pandemic (5, 6).

## Methods

To provide a summary of the potential roles and responsibilities of pharmacists during the COVID-19 pandemic, published data from scholarly articles, organizations, and stakeholders were reviewed. Literature was searched by four independent reviewers (THM, AL, AA, YHK) using various electronic databases from inception to July 2020. The end date of the review time generally coincides with the generally accepted end of the first wave of COVID-19 in the Northern Hemisphere.

### Selection Criteria for Studies

The current review describes the various roles of pharmacists that can be performed during the COVID-19 pandemic to leverage all possible resources in the best interest of patient care and management. Moreover, data from studies providing prospective implications of pharmacy services in the management of COVID-19 pandemic were also included to give insight of the possible roles of pharmacists in combating COVID-19. Relevant data was collected via electronic search of different scientific sources including PubMed (https://www.ncbi.nlm.nih.gov/pubmed), Science Direct (https://www.sciencedirect.com/), Google Scholar (https://scholar.google.com/), Scientific Electronic Library Online (SciELO) (http://www.scielo.org/), Cochrane Library (https://www.cochranelibrary.com/), and Web of Science (http://www.webofknowledge.com/). The study databases were selected on account of their score for health and pharmaceutical journals. These databases covered articles of peer-reviewed journals, books, and supplementary reports covering multilevel engagement of pharmacists in COVID-19.

### Search Strategies

The search strategies utilized a combination of the following terms: “COVID-19,” “Corona virus disease,” “pharmacist,” “community pharmacist,” “industrial pharmacist,” “hospital pharmacist,” “drug regulatory authorities,” “regulatory pharmacists,” “pharmaceutical industry,” “pharmacy department,” “SARS-CoV-2,” “pharmacy management,” “pharmaceutical care,” “clinical pharmacist,” “pandemic,” and “outbreak.”

### Inclusion/Exclusion Criteria

Studies on the functions and roles of pharmacists during the COVID-19 pandemic published during the period from inception to July 2020 were included in this review. Moreover, this review also included the studies describing the various fundamental responsibilities of pharmacists which can be utilized during the ongoing health crises. Only research studies, review articles, case studies, books, short reports, position papers, perspectives, organizational recommendations, and authentic guidelines on management and containment of COVID-19 were considered. Abstracts, scientific correspondence, posters, advertisements, thesis, web pages, and news were excluded. The studies published in a language other than English were not included in this review.

### Data Extraction

Following the introductory search, retrieved articles were imported to EndNote X7 to remove the duplications. The eligibility of the each study was assessed by all authors through screening of the title and abstract. After an initial screening, a full-text evaluation was carried out for the final selection of articles. Any disagreement regarding the suitability of studies among authors was resolved through mutual agreement and discussion. Only studies for which consent was provided by all authors were included in the manuscript. The studies related to the potential role of pharmacists for COVID-19 were selected and carefully analyzed for this review. With the information assembled through these studies, the difference between the traditional and the expanded role of pharmacists was pointed out. Major suggestions are offered on how to utilize the contemporary roles of pharmacists to effectively employ all available resources thus easing burden on other healthcare professionals.

## Role of Pharmacists in Previous Outbreaks

Pharmacists have been actively involved in various infectious disease outbreaks. During epidemics, the role of pharmacists broadened from routine duties to preventive activities such as disease monitoring and surveillance, immunizations, and diagnostic testing (7). Pharmacist associations around the globe have played a crucial role in pandemics to plan beforehand in order to leverage all the resources to their maximum extent (8). According to the Canadian Pharmacists Association (9), the role of pharmacists was designed in pandemic preparedness plan for combating H1N1 influenza (9). To combat the shortage of antivirals, pharmacists compounded oseltamivir in the hospital pharmacy during the influenza (H1N1) pandemic (10). Pharmacists played their role in effectively promoting an alternative method of supply and dispensing of antivirals from public stockpiles during the flu pandemic (11). Nigerian pharmacists helped during the Ebola outbreak by promoting infection control measures and educating the public on how to avoid the disease spread (12). Pharmaceutical care, medication therapy, infection control, and immunization are among the top listed duties delivered by the pharmacists during the Ebola epidemics (13). Effective communication between health departments and community pharmacies was proved to be an effective response to the influenza pandemic (7).

The role of pharmacists is well-appreciated during the outbreaks of vaccine-preventable diseases. The education of patients and the public regarding the importance of vaccines resulted in increased vaccination rates (14). Pharmacists played a valuable role in the protection of high-risk individuals through vaccination programs (15).

Pharmacists are known for reducing the work load of public health professionals during the measles outbreak. Being an easily available, accessible, and trustworthy professional, pharmacist ran vaccination plans and increased the vaccine awareness which ultimately resulted in vaccination acceptance rates (16). Pharmacists provided patient-centered therapy with stringent infection control measures during the severe acute respiratory syndrome (SARS) outbreak. They delivered the medicines not only in wards but also in quarantine areas. Pharmacists have proved themselves as a valuable member of the team in health crisis by providing information services and patient care (16, 17).

**Figure 1:**
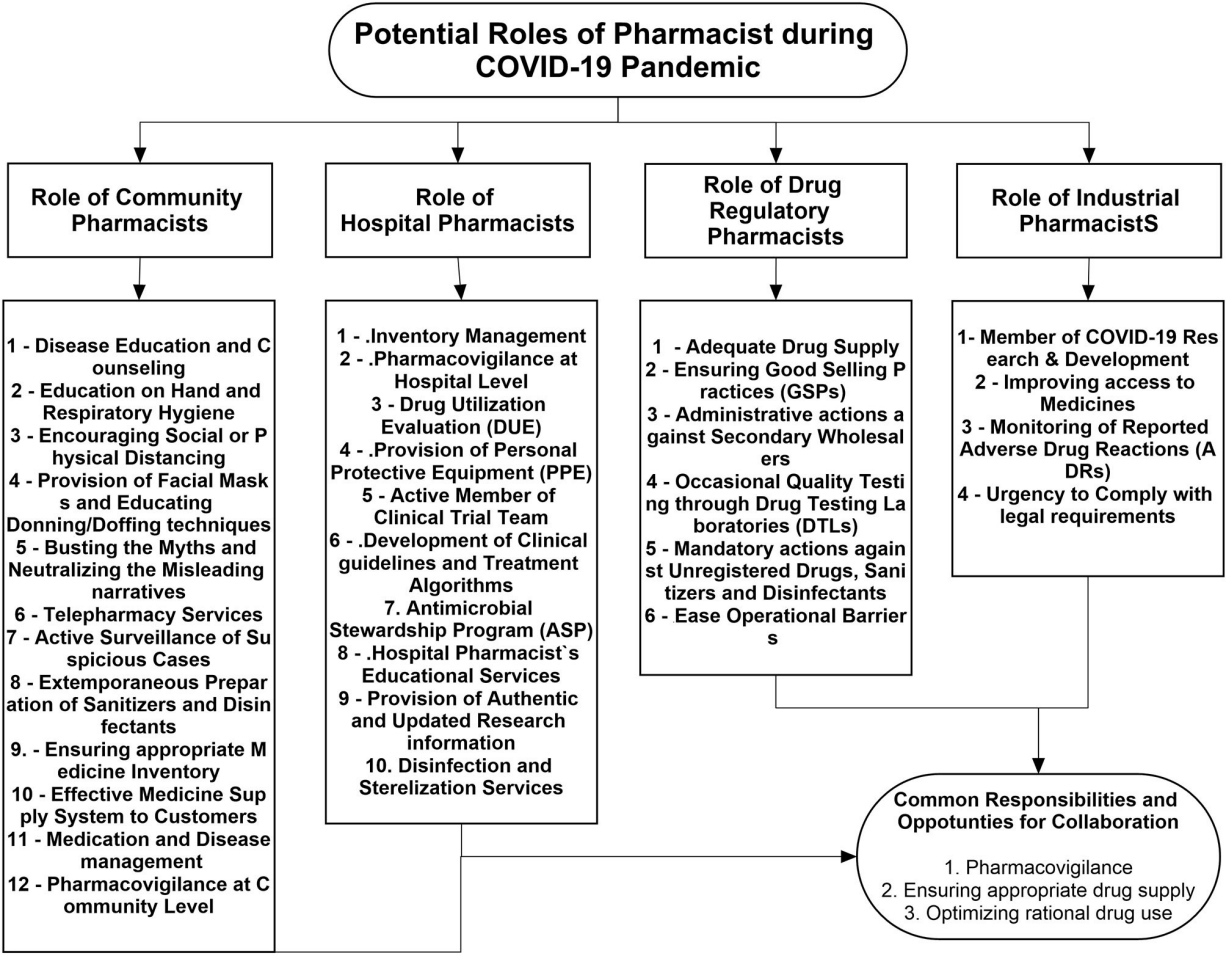
Potential role of pharmacist during the COVID-19 pandemic.

Keeping in view the previous contributions and current extended pharmacy services, pharmacists can be involved at different levels in disease control and prevention, patient care, and treatment during the COVID-19 turmoil. Figure 1 describes the potential contributions of pharmacists working at different levels of the healthcare system.

### Role of Community Pharmacists

Community pharmacies have remunerated their roles from traditional dispensing to more comprehensive clinical services in recent years. Community pharmacies are considered the first entry point in both outbreak-affected and unaffected areas during the COVID-19 pandemic (18), where pharmacists are most accessible and underutilized healthcare professionals to cope with COVID-19 havocs (6, 19). Community pharmacists can provide breadth of public health and clinical pharmacy services during the ongoing pandemic to empower people to self-manage their health. Following are the potential roles of the pharmacist at the community level to serve the public in the current circumstances.

#### Disease Education and Counseling

The COVID-19 pandemic is accompanied by several misleading narratives which raised confusions and uncertainties among the general public (20). The importance of public awareness and education for COVID-19 cannot be disregarded during the current pandemic. Pharmacists being frontline healthcare providers can guide people regarding the disease, its causes, and routes of transmission, thus assisting to neutralize the confusions in the general community (21). Counseling of patients regarding the onset of sign and symptoms after contracting the virus is very crucial as the patient can remain asymptomatic for 2–14 days following virus exposure (22). The median incubation time for coronavirus is 5.1 days, suggesting the appropriateness of quarantine time of 14 days (23). Pharmacists should be well aware and provide sound advice to the public about the clinical manifestations of the disease. Moreover, they can provide advice to suspects on whether there is a need to be quarantined or not. Since the virus can spread through airborne droplets, coming in contact with infected persons, and by touching contaminated material surfaces (24), the disease education carries utmost importance to halt the transmission chain (25).

With the rise in the number of COVID-19 cases, there is unprecedented burden on the healthcare system overwhelming hospitals and healthcare facilities (22). In this critical time, pharmacists can provide proper disease education at the community level to avoid the unnecessary scares (3). Symptoms-related counseling will help the patient to understand the point or time when medical attention is needed. As most of the suspects are visiting the emergency department even due to cough or flu which is adding unnecessary workload on the hospitals (26), community pharmacists can educate the public about the differentiating symptoms, thereby reducing the unnecessary visits to the emergency departments.

Likewise, during the current pandemic, self-medication is becoming prominent due to amplified information on drugs floating on social media and news channels. Drug repurposing is improving the clinical conditions of patients but at the same time portending substantial risks of self-medication (27). Various reports have indicated chloroquine poisoning and shortage following the announcements related to its effectiveness on electronic media (28, 29). Community pharmacists must observe any kind of unusual drug use among the public or any irrational prescribing pattern among physicians in their localities.

#### Education on Hand and Respiratory Hygiene

Pharmacists can utilize several methods of education to enhance the public understanding of disease (30). Educational presentations in video formats, posters, flyers, or hangings could prove very impactful in educating the masses coming to the pharmacy. Pharmacists can arrange hand sanitizers at all patient counters and should practically demonstrate the correct method of hand sanitization. Similarly, considerable attention should also be paid to good hygiene practices among pharmacy staff. Isolation of pharmacy staff from the customers through a glass screen or polythene material would not only give a message to the customers about the importance of physical distancing but will also ensure the protection of the staff. Appropriate practical gesture will have long-lasting impact on customers about the seriousness of good hygiene (23).

#### Encouraging Social or Physical Distancing

Teaching people about social distancing would be easier if all the pharmacy staff is also practically demonstrating it to the customers. There should be at least 1 m (3 ft) distance between every pharmacy personnel. The floor of the pharmacy can be marked with the signs of 1 m distance to avoid any close contact among the customers. This method will provide actual sense of physical distancing to the customers. The minimum safe distance to avoid viral contract should be displayed at every counter. Customers should be encouraged to maintain at least minimum distance while handling the routine matters. Several studies have suggested to maintain at least 2 m (6 ft) distance to avoid any viral contraction (31–35). The public should appropriately be counseled to avoid any contact with the surfaces frequently used by the people such as door knobs or handles, and in case if they have come in contact with such surfaces, they should follow immediate hand wash technique and should avoid touching their face. Since pharmacies have become frequently visited places during the pandemic, effective utilization of an educational tool will help to provide authentic and useful information to the general public (36).

#### Provision of Facial Masks and Educating on Donning/Doffing Techniques

The use of masks is a basic protective measure to avoid viral contract from the patients (37). However, its effectiveness can only be ensured following the appropriate methods of use. Pharmacies are authentic selling points for masks and can be served as an educational platform for the public to get awareness regarding the types of masks and their correct methods of use. Incorrect use of masks and inauthentic brands will not only increase the risks of disease contraction but will also pose economic burden to the public and may lead to shortage of masks for high-risk users (38). Pharmacists should use visual aids to educate the public about the donning and doffing techniques and should ensure that people are using authentic masks dedicated for viral protection. Correct donning and doffing of masks and their appropriate disposals are among most effective measures stated by various health authorities around the globe (37).

#### Busting the Myths and Neutralizing the Misleading Narratives

The COVID-19 pandemic is accompanied by rapidly prevailing myths on treatment and prevention. Moreover, several conspiracy theories and misleading narratives have sprouted and proliferated in many countries, posing substantial challenges for health officials to contain the disease. Since community pharmacists are considered trusted professionals, they can play a pivotal role in busting these myths by providing reliable information to the public. Pharmacies can make pamphlets on prevailing myths and can distribute to each customer attending the premises. They may ask questions to the customers in order to ascertain their perception toward the disease and can clarify any confusion. The WHO has issued various videos and images to eradicate the myths and the same can be utilized in the pharmacy premises (39). Moreover, it is pertinent to mention that every news channel or agency is efficiently engaged to break any new findings or studies related to the treatment and prevention of COVID-19. Such news reports may influence the people who are searching for appropriate measures to save themselves from the virus. Pharmacists at the community level can provide timely intervention to bust such claims and prevent the public from self-medicating. Any sort of self-medication during the current pandemic can be devastating and may aggravate the ongoing health crises for which none of the country is readily prepared.

#### Telepharmacy Services

COVID-19 has changed the use of informational technology in the healthcare system. Telemedicine provides electronic consultations and has reduced the risk of transmission by reducing in-person contact among people. Telepharmacy is one of the practical aspects of telemedicine that refers to providing pharmaceutical services within the scope of a pharmacist's responsibilities, with a temporal and spatial distance between patients as the consumers of health services and healthcare providers. This service will not only be useful for COVID-19 patients but also for chronic patients and the general community experiencing the restricted movements amid lockdown. Moreover, telepharmacy will also reduce the volume of patients seeking care at health facilities (40). Though the telepharmacy program would not solve all the health problems, it is well-suited as a solution to make effective connection between pharmacists, patients, and other healthcare professionals. Moreover, community pharmacists can consider this service to follow COVID-19 patients after discharge to ascertain the recovery pattern. For routine chronic patients, pharmacies can use their webpages, message services, or social networking links to respond to the queries.

#### Active Surveillance of Suspicious Cases

Early detection and referral of suspected cases are vital to prevent large-scale community transmission. Community pharmacists, therefore, must remain very vigilant and be able to screen the patients for necessary referrals. The Centers for Disease Control and Prevention (CDC) and the International Pharmaceutical Federation (FIP) have provided guidelines for the investigation of suspected COVID-19 cases (21). There are several other risk assessment scales available to identify the suspected cases such as CDC's coronavirus self-checker (41). Pharmacists should arrange sensitive thermometers at the pharmacy to identify the suspects. Any suspected case must immediately be notified to the designated health authorities in order to avoid any further disease spillover. Since most of the people are hiding their symptoms due to the fear of being quarantined or due to the phenomenon called disease denial, pharmacist can be a facilitator for national disease surveillance unit. Community pharmacies along with educating the public can play a crucial role in case identification and reporting.

#### Extemporaneous Preparation of Sanitizers and Disinfectants

The COVID-19 pandemic has caused shortage of hand sanitizers in many developing and developed country, either due to increased consumption or disruption of raw material supply. Community pharmacists can employ their expertise of compounding and ensure the availability of hand sanitizers and disinfectants all the time at an affordable cost. The WHO recommended the use of ethanol (80%) and isopropyl alcohol (75%) in all kinds of hand rub formulations (23). Pharmacists can also prepare disinfectants according to national legal provisions. Moreover, all surfaces of pharmacies should also be disinfected. A simple formulation of 10 ml bleach in 990 ml water can be used as an effective disinfecting solution (31).

#### Ensuring Appropriate Medicine Inventory

During these unprecedented times, the global drug supply could severely get impacted by this pandemic, and the results of this shortage could be catastrophic and may last for an extended period, primarily due to the global economic disruption at unprecedented speed and scale. It must be noted that drug shortage could lead to serious consequences when it comes to patient outcomes (42). Pharmacists can play an important role in the mitigation of emerging drug shortages related to the pandemic. Pharmacists should aim at procuring and stocking the right amount of medicine to guarantee the supply round the clock. The regulatory body of pharmacy or the whole department should work in collaboration and devise shortage and mitigation plan beforehand (43). Drug demand analysis to identify medications of interest should also be conducted in a parallel manner (44). One pharmacist can be designated specifically for procuring to ensure the supply of medicine and to avoid any possible shortage. Meanwhile, they should also use their pharmacologic and pharmacokinetic knowledge to design algorithms to successfully implement medication-sparing strategies (44). Vigilant observation of any medicine's unusual selling trend and timely reporting can prevent drug shortage. Pharmacists must use their compounding abilities and utilize alternate ingredients to ensure proper supply of medications, sanitizers, and disinfectants (43). Since ensuring the availability of all medicines during the current state of unrest becomes quite difficult, community pharmacies can make a priority list according to the regional need and should ensure the availability of these medicines, particularly those required for COVID-19 management (44). Moreover, the pharmacist is able to navigate alongside other members of the healthcare team alternative therapeutic options until the shortages are resolved.

#### Effective Medicine Supply System to Customers

In order to ensure appropriate supply of medications especially in small towns where local pharmacies may have closed, community pharmacists can arrange home deliveries or an electronic prescription refill system. Home delivery services will prove to be very helpful for people in quarantine as well as for those with weak immunity, i.e., the elders (25). Pharmacists can be authorized by governing bodies to use their judgment to refill prescription for at least 30 days to avoid unnecessary patient visits. Similar practice has been adopted in the USA during the time of Hurricane Katrina (45). In order to avoid pharmacy visits, early refills for maintenance medicines would also be beneficial for the patients with chronic illnesses (45). Moreover, pharmacists should also ensure that medicines are being sold for the purpose of use rather than for stocking.

#### Medication and Disease Management

It is a critical time to convert the traditional role of pharmacists into the extended role to allow them provisional prescribing of medications in collaboration with a physician within some jurisdiction (19). This function would be critical in areas where healthcare providers are busy with COVID-19 management and deployed from their routine practice sites to other areas. It will ultimately increase the dependence of these healthcare professionals on pharmacists as the medication experts on the team (44). Patients with chronic diseases can benefit from this service where they do not have to visit the hospital for their prescription refill (45). Moreover, pharmacists can arrange on-call meetings with physicians to seek any guidance regarding prescription refills. These extended pharmacy services (EPS) will possibly reduce the mobility of patients with chronic illnesses. It must be noted that chronic patients have portended higher mortality rate during the COVID-19 pandemic. Moreover, recent data has indicated the high ability of chronic patients to contract the virus and to have severe symptoms of COVID-19 (46). Therapeutic switching is a major challenge for clinicians due to drug shortage during the pandemic. Pharmacists should intimate local clinicians regarding the availability of therapeutic alternatives. Examples here include the switching between the intravenous analgesic fentanyl to remifentanil. Since the supply of intravenous medications is substantially high during the pandemic, pharmacists should educate other members of the healthcare team about the use of adjuvant medications such as oral and transdermal formulations.

#### Pharmacovigilance at the Community Level

Since the healthcare system is overwhelmed with the increasing number of COVID-19 cases, any drug-related problems (DRPs) might easily be neglected by healthcare professionals. These DRPs are either related to the ongoing use of chronic medications or self-medication and may associate with adverse outcomes. Pharmacists must keep a vigilant observation on the safety profile of these drugs. As most of the healthcare authorities are engaged in containing the virus, any untoward effect of the drug will get unnoticed (25). It is a fundamental responsibility of community pharmacists to ensure the safe use of drugs, particularly among the chronic patients. Moreover, community pharmacists should efficiently monitor the potential side effects of repurposed drugs employed in the prevention and treatment of COVID-19.

### Role of Hospital Pharmacists

In public health emergencies, pharmacists play a distinguished role in reducing the burden of disease. Hospital pharmacists provide pharmaceutical care and services to both in-patient and out-patient (7). During the COVID-19 pandemic, their duties expanded from routine activities to the focused care for COVID-19 hospitalized patients. Pharmacists became an important part of the medical team to improve the therapeutic outcomes and ultimately the pandemic control (47). They also ensure the adequate supply and stock of requisite drugs and other medical products in accordance to the patients' demand (48).

#### Inventory Management

Hospital pharmacists are primarily responsible to ensure the timely provision of medications to the patients (49). Pharmacists can play a vital role to identify and alleviate possibilities of drug shortages. Drug shortages in the current scenario can compromise or adversely affect the patient medication therapy. Moreover, pharmacists should identify the factors in the supply chain contributing to the drug shortage. The pharmacy staff can contact pharmaceutical manufacturers, distributors, community pharmacies, and the regulatory agencies to inquire about the cause and duration of the shortage (50). Due to limited trade and closure of various pharmaceutical plants, disruptions in the pharmaceutical supply chain are observed resulting in increased prices and drug shortages globally (51). In this inevitable situation, pharmacists can find therapeutic alternatives to avoid any hindrance in the provision of therapy. They should develop a proactive attitude through quantitative assessment of the inventory and estimation of the drug shortage period. Alternatives should be inventoried to ensure sufficient supply to meet the increasing demand (52). Moreover, provision of drugs to prioritized patients is the need of the hour. It would be beneficial to reserve repurposed drugs for COVID-19 patients (53). Hospital pharmacists can assist clinicians in drug switching and adjunctive therapy to cope with the drug shortages.

#### Pharmacovigilance at the Hospital Level

Effective pharmacovigilance results in reduced cost of care while improving therapeutic outcomes, which are the need of the hour during the ongoing pandemic (54). Currently, numerous drugs are being tested for COVID-19 and few of them are linked to serious adverse effects. Chloroquine and its derivative hydroxychloroquine pose risks of QT prolongation and require caution among patients with G6PD deficiency and diabetes. Lopinavir and ritonavir are associated with the risks of cardiac arrhythmias due to QT prolongation, and careful monitoring is required among patients with hepatic problems. Corticosteroids are considered for patients with respiratory distress syndrome or refractory shock and are not recommended for viral pneumonia (55). Baricitinib should be used with extreme caution in susceptible patients with ongoing pneumonia associated with SARS-CoV-2 (56). During the current phase of drug repurposing, the hospital pharmacists are keenly monitoring drug safety by detecting, investigating, and reporting drug-related problems among COVID-19 patients (57). Moreover, hospital pharmacists should review safety data of published studies which are desperately needed by healthcare professionals. Despite the unprecedented global challenge posed by the COVID-19 pandemic, the importance of patient safety should not be disregarded.

#### Drug Utilization Evaluation (DUE)

Drug repurposing for COVID-19 is improving the clinical conditions of patients but at the same time posing substantial risks of drug-related problems. The use of these drugs is subjected to careful assessment only if the desired effects overshadow the risks. Pharmacists provide accurate clinical information to the healthcare professionals regarding the drug safety, interactions, and adverse effects (53). Optimizing the rational use of repurposed drugs is the need of the hour. A study from China indicated that the use of ribavirin for COVID-19 widely varies in hospital in terms of duration and timing of treatment (58). Pharmacists should ensure the appropriate use of these medications in the hospital, particularly in the vulnerable population such as the elderly, immune-compromised patients, and pregnant women. These population should be considered for targeted drug use evaluation (59). Moreover, routine DUE should not be neglected during the current pandemic.

#### Active Member of the Clinical Trial Team

Since most of the hospitals are conducting clinical trials on the treatment of COVID-19, pharmacists can improvise their roles in the provision of the right administration of drugs and with appropriate documentation. Moreover, pharmacist involvement in these trials will strengthen the findings and aid to minimize the potential bias. The primary areas in which pharmacists can work include safety and efficacy evaluation, provision of drug or placebo, and follow-up of patients to ensure optimal therapy (49, 50). Being a core member of the antimicrobial stewardship team, pharmacists can facilitate the use of investigational drugs, where the antivirals with established efficacy are being evaluated for prophylaxis and treatment of COVID-19 (53).

#### Development of Clinical Guidelines and Treatment Algorithms

In the absence of specific treatments for COVID-19, there is a dire need of clinical guidelines and treatment algorithm to manage the patients. Pharmacists, physicians, and other healthcare professionals can work together to develop these guidelines. Since clinical information is rapidly changing and evolving with ongoing research, these guidelines must follow evidence-based practice. Several organizations have provided the stepwise management of patients with COVID-19 (46, 50, 60–63). The primary care consists of symptomatic management and oxygen support (64). Pharmacists can play a vital role in the formulation of dosing regimen and monitoring of safety and efficacy of the drugs (65). The FIP has also formulated a list of medicines being used in COVID-19 to help pharmacists around the globe (50). Hospital pharmacists can prepare dosage guidelines, precautionary notes, and list of potential drug interactions, adverse drug reactions, and contraindications.

#### Antimicrobial Stewardship Program

Increasing antimicrobial resistance is an important public health problem. Pharmacists play an integral role in the team to implement antimicrobial stewardship programs (ASPs) along with physicians and nurses. The program typically focuses on the use of evidence-based data and monitors the antibiotic consumption and sensitivity patterns in the patient population to highlight and promote rational antibiotic prescribing (66). The pharmacists provide individualized patient treatments, improve therapeutic outcomes, and contribute in the rational prescribing of antibiotics as per antimicrobial stewardship guidelines, ultimately reducing the development of resistance (67). During the COVID-19 pandemic, implementation of ASPs has assisted pharmacists in the formulation of treatment protocols for repurposed antiviral drugs and improving therapeutic interventions for patients (68). ASPs have a potential role as the gatekeeper for appropriate use of COVID-19 drugs in order to optimize patient‘s selection and to minimize antibiotic misuse. Moreover, ASPs will also assist to curb the potential threats of shortage of repurposed drugs, i.e., hydroxychloroquine, especially for patients with prime indications such as rheumatological disorders. Formulary restrictions and preauthorization through ASPs will ensure proper allocation of medications for patients in high need. Since there is a high concern that antimicrobials may be overused among COVID-19 patients, hospital pharmacists should focus their efforts to establish an effective ASP in collaboration with other healthcare professionals (69).

#### Hospital Pharmacists' Educational Services

Hospital pharmacists interact with patients and their caregivers at the time of hospital discharge to provide advice on the appropriate use of medications. This service can be utilized to educate patients regarding precautionary measures in order to prevent the acquisition of infection. As family members of COVID-19 patients are at high risk of contracting the infection, their education and awareness carries paramount importance. Hospital pharmacists can continue their clinical services for chronic patients through telepharmacy in order to avoid the contact of the patients to the hospitals (49). Pharmacists can also provide education to nurses and paramedic staff on the appropriate drug administration, use of protective equipment, and reporting of adverse events. These educational series will not only optimize patient care but will also reduce the burden on clinicians working at the frontline during the pandemic. Hospital pharmacists should ensure that nurses and allied health staff are well-equipped with the necessary knowledge and skills required to combat COVID-19. Pharmacist can educate hospital staff regarding the standard operating procedures to deal with the patient's samples, belongings, and waste material (53). In the setting of the current pandemic, ASPs can be actively involved in educating providers on local COVID-19 treatment protocols, especially if ASPs are involved in developing treatment guidelines. As discussed earlier, education is a core activity of ASPs and can be utilized to increase the awareness among other healthcare professionals regarding the drug toxicities associated with COVID-19 treatments.

#### Provision of Authentic and Updated Research Data

As most of the physicians and nursing staff are overburdened amid unprecedented cases, pharmacists could act as authenticated and updated source of information. The research on COVID-19 is quite dynamic as numerous new findings are originating every day. As hospital pharmacists are also experts in research interpretation, they can timely disseminate information on the recent advancements in the COVID-19 management and prevention. Moreover, pharmacists can communicate to healthcare providers regarding potential medication-related problems (MRPs) of repurposed drugs in order to ensure the drug safety and optimal therapy (53).

#### Disinfection and Sterilization Services

Hospital pharmacists are responsible and strictly advised to adhere to the regulations for infection prevention and control in hospitals and medical institutes. Since COVID-19 demonstrated rapid transmission, disinfection, and sterilization services are of utmost importance to contain the virus. Hospital pharmacists along with the hospital's infection prevention team should ensure the disinfection of all surfaces within the hospital premises (49, 50, 53). These safety measures aid to protect both the pharmacy staff and patients from infections (65).

### Role of Industrial Pharmacists

The COVID-19 pandemic caused increased demand of drugs, surgical supplies, personal protective equipments (PPEs), and supportive care appliances. With many countries introducing lockdowns and travel restrictions, the global network for manufacturing and delivering medicines is widely affected. Pharmaceutical industries are experiencing major challenges in securing deliveries of medicines, not only for COVID-19 but also for other diseases. In this context, the responsibilities of industrial pharmacists have much increased. The continuous and timely production of key drugs being utilized during the current pandemic is of paramount importance and contributes substantially to alleviate the disease burden (49, 50). Industrial pharmacists are key players during the current battle against the virus and can ensure following tasks.

#### Member of COVID-19 Research and Development

The worldwide COVID-19 outbreak has highlighted the importance of the timely development of not only vaccines but also broad-spectrum antiviral drugs (70). Currently, most of the pharmaceutical organizations are engaged to sponsor or conduct clinical trials on vaccines and drugs (71). Numerous research experiments and trials are undergoing to accelerate vaccine and drug development (72). Industrial pharmacists should ensure the appropriate and safe use of medications during the clinical trials. Moreover, pharmacists working in industries are also responsible to ensure the ethical aspect and effective resource utilization during clinical research conducted by the industry (49, 50).

#### Improving Access to Medicines

Pharmacists working in the industries are well aware of the significance of timely distribution of medications to patients, particularly life-saving and essential drugs during the current emergency circumstances (73). Unavailability of medicines creates frustration for everyone including pharmacists, physicians, nurses, and patients. Various factors such as trade restrictions, strict registration, and regulatory compliance may result in drug shortages (52). Industrial pharmacists must be fully prepared and should develop a contingency plan to avoid any sort of shortages. However, various health regulatory agencies have spared and relaxed the manufacturers from the fulfillment of conventional regulations and granted the priority approvals for the repurposed and experimental drugs (73–75). In such circumstances, the responsibilities of pharmacists are enhanced to ensure the standards, quality, and ethics.

#### Monitoring of Reported Adverse Drug Reactions (ADRs)

Industrial pharmacists are vigilantly working in the pharmacovigilance centers as the qualified person for pharmacovigilance (QPPV). They receive information from the healthcare authorities and other pharmacists dealing with COVID-19 patients about any adverse events of the proprietary drug. Moreover, it is also the fundamental responsibility of the pharmacist to update these events in the National Pharmacovigilance Database. Pharmaceutical organizations producing investigational drugs should ensure safety reporting according to national legal requirements. Industrial pharmacists are also responsible to collect safety data of their drugs by all means including personnel visit, email, phone, or fax. Pharmacists should develop a smooth and convenient reporting tool for the adverse events (76). Moreover, any updated information regarding the safety and efficacy of the drug should be provided to the healthcare professionals in a timely manner. Marketing departments of the pharmaceutical organizations can be an important source of feedback from the healthcare professionals.

#### Urgency to Comply With Legal Requirements

The impact of COVID-19 on pharmaceutical companies has been unique, as organizations had setup emergency management systems to continue their operations. Moreover, healthcare professionals and the general public are also expecting significant contribution of industries against COVID-19. During these unprecedented times, industrial pharmacists need to adopt proactive and active measures to fulfill the legal requirements of production and supply. Since regulatory authorities in many countries provided legal flexibilities (77), pharmacists should ensure that drug supply should not be disrupted merely due to legal procedures.

### Role of Drug Regulatory and Administrative Pharmacists

The responsibilities of drug regulatory and administrative authorities have increased amid the sharp increase in the demand of medications and drug repurposing applications. A quick response from these authorities is of utmost importance to ensure the appropriate use of medications in the current state of unrest. They can ensure that pharmacists performing in community pharmacies, hospitals, clinics, or in industrial sectors are fully equipped and authorized to respond to the COVID-19 emergency plan (43, 78). A rapid increase in drug demand requires vigilant monitoring of supply, procurement, and storage. The regulatory department can ensure appropriate, timely, and necessary availability of the drugs at the points in high demand. Following is the list of domains in which drug regulatory authorities can effectively respond.

#### Adequate Drug Supply

Since the COVID-19 pandemic continues to put strain on the healthcare system, pharmacists at administrative levels can develop a COVID-19 emergency preparedness plan to mitigate the disruptions in supply which could lead to drug shortages (21). Specific committees can be designed to estimate the quantities of essential patient care medications, equipment, and PPE at varying patient care facilities. Such kind of forecasting and planning would help in conserving medicines, equipment, and supplies before they go black in the market. In addition to the forecasting analysis, quick and smart response is the need of the hour. The administration can ask federal health authorities to safeguard the drug supply which will help in maintaining transparency in the supply chain (79). Moreover, reporting of the causes of drug shortage and its expected duration will aid in institutional planning to manage the alternative sources of drug procurements. Drug administrative authorities should highlight alternative vendors to make sure the availability of life-saving drugs. Priority supplies to tertiary care hospitals particularly those dealing with COVID-19 patients will aid to avoid any drug shortages in these facilities.

#### Ensuring Good Selling Practices (GSPs)

Pharmacists are well-positioned to reduce risks of further medication shortages arising from COVID-19 by reassuring patients and members of the public of the continued availability of OTC and prescription medications based on rational levels of demand and implementing policies to prevent unnecessary stockpiling (45). Recent reports have indicated the shortage of chloroquine (CQ) and hydroxychloroquine (HCQ) which would create problems for the patients with systematic lupus erythematosus and rheumatological and dermatological disorders (80). The probable causes of shortage are stocking of drugs by patients, hospital administration, drug retailers, and physicians (81). Drug regulatory pharmacists must observe any unusual sale of a particular medicine or equipment in order to make strict oversight against parties involved in price gouging and those taking advantage of the heightened demand for supplies. Similarly, pharmacists at the administrative levels should make sure the pharmaceutical companies and other stakeholders realize their duty and make sure the availability of the drugs to those who need them the most. A combined sensible effort of community pharmacists along with drug regulatory and administrative authorities could definitely help through this time of crisis.

#### Administrative Actions Against Secondary Wholesalers

In the best interest of patient care, pharmacists and clinicians must be able to access the medication and supplies they need during the current outbreak. Personal protective equipment commonly employed in healthcare facilities are now a scarce commodity. The heightened price of PPE including masks, gloves, respirators, goggles, face shields, and gowns is recorded in the USA and in many other countries around the globe (82). The demand for specific PPE used in response to COVID-19 has increased about 1,000- to 2,000-folds. Moreover, N95 respirators are also experiencing the largest constraints. The procurement of sanitizer has largely been affected both in terms of purchasing and delivery (83). These issues must be addressed in haste to quell the further deterioration of the drug supply system. We suggest the following actions that must be taken at the administration level to mitigate the stock hoarding and price gouging practices during a pandemic:

- Active surveillance to identify the secondary wholesalers involved in stockpiling and price surging activities.
- Increase supply chain security by working with manufacturers.
- Shortage of personal protective equipment (PPE) can be prevented at the federal level by identifying and reporting the agents involve in hoarding and should be dealt with an iron hand (79). Moreover, guidance in making PPE from alternative resources can be provided to pharmacists that can effectively prevent their shortage.
- Monitoring of purchasing trends by the pharmacies and frequent follow-up to ensure the availability of the stock in the premises.
- Administrative authorities should contact reputable manufacturers and supplier to ramp up production and meet the increased demand needs.

#### Occasional Quality Testing Through Drug Testing Laboratories (DTLs)

Relaxing regulatory requirements can effectively ease the manufacturers to increase the supply of medicine and equipment needed during the current pandemic. Considering the occasional testing of medications through drug testing laboratories will shorten the time span of drug availability in the market. Such sort of regulatory relaxations will be beneficial for alleviating the shortage of medicines which are being used in supportive care of COVID-19 (79). However, additional caution must be practiced for the drugs for which provisional quality approval is granted. It must be noted that relaxation of regulatory conditions should not compromise the quality of production and supply.

#### Mandatory Actions Against Unregistered Drugs, Sanitizers, and Disinfectants

As part of the surveillance team and contingency planning, administration can stem out the false and misleading claims about products purporting to treat or prevent COVID-19 (79, 84). Drug inspectors and drug monitoring teams should be authorized to seize and impede the sale of any unregistered drug and report bad actors. Similarly, shortage of hand sanitizers and disinfectant can also be dealt in same way to stop their hoarding and stockpiling (79). Meanwhile, proper training can be provided to all pharmacists working in any sector for small-scale manufacturing of sanitizers and disinfectants from alternative sources.

#### Ease Operational Barriers

Easing operational barriers for pharmacists working in any health facility will help in their effective engagement in COVID-19 response (85). Drug administrative authorities can allow a grace period in the renewal of licensures during emergency period. They should also waive some restrictions of good manufacturing practices thus making sure the timely availability of the medicines.

## Interprofessional Collaboration in The Healthcare System

**Figure 2:**
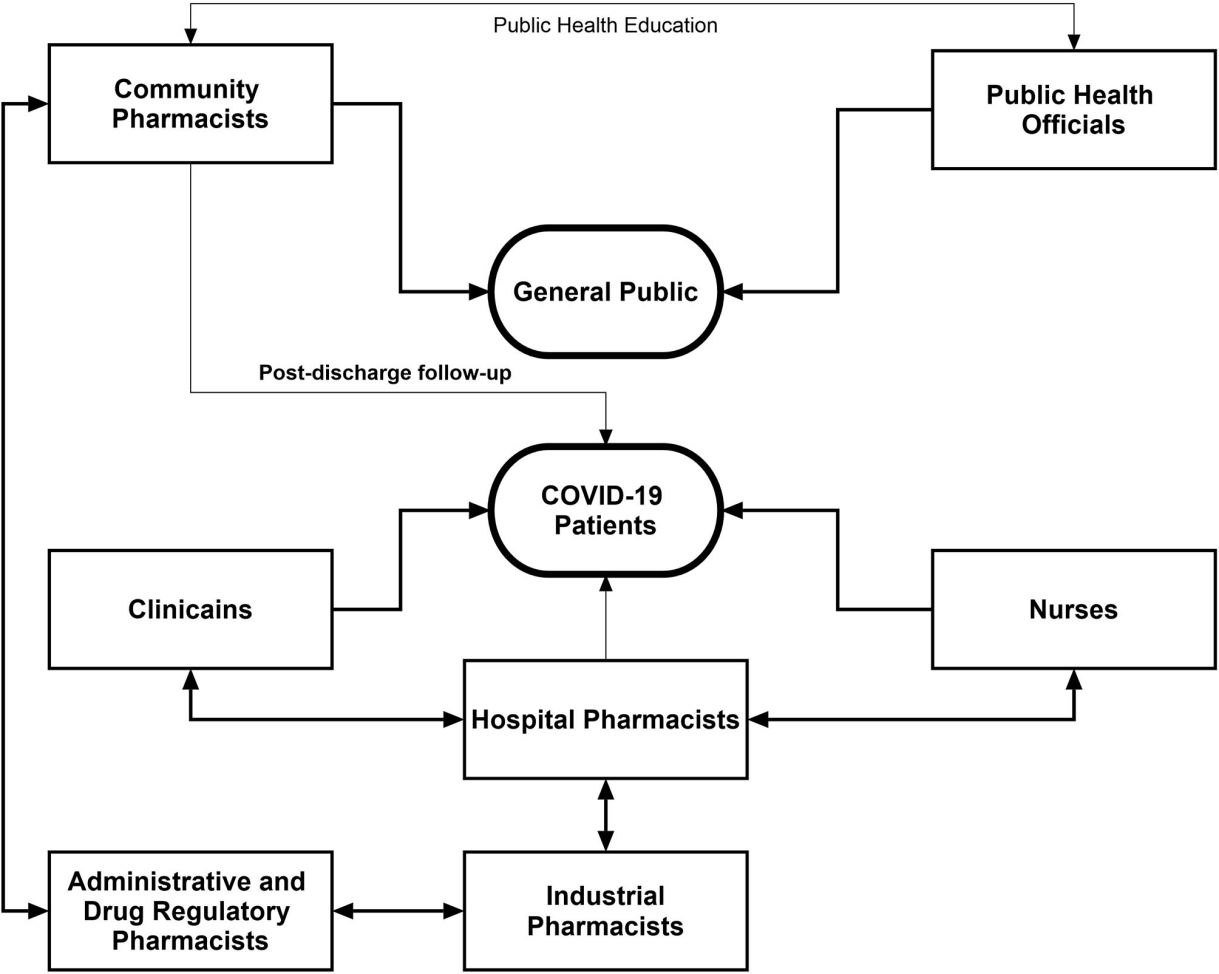
Interprofessional collaborative framework of pharmacists with other healthcare professionals during the COVID-19 pandemic.

It is important to recognize the pharmacists' engagements through interprofessional collaborations, which involves pharmacists and other healthcare professionals from various disciplines working together with shared goals, mutual interest, respect, and understanding about each other's roles, along with the acceptance that patients are team members (86). It is pertinent to mention that there is a substantial relationship between the extent of interprofessional collaboration and patient safety (87). We believe that the battle against COVID-19 can only be won through collaborative maneuvers. Figure 2 describes the summary of the collaborative framework of different healthcare professionals to combat the pandemic. Community pharmacists can assist drug regulatory authorities in identifying the unusual practices such as irrational use, stocking, and misconception among the general public related to the repurposed drugs. Keeping in view the easy accessibility of community pharmacists, they can work with public health officials to ensure that the population is complying with the preventive measures and to neutralize the myths related to COVID-19. The public health department should consider the potential of pharmacists in this regard.

Community pharmacists along with clinicians can effectively arrange the postdischarge follow-up of COVID-19 patients which could provide greater insight into the recovery pattern of such patients. Hospital pharmacists can optimize patient outcomes through working collaboratively within multidisciplinary teams to achieve the responsible use of medicines. In addition, hospital pharmacists should aid overburdened clinicians and nurses through medication reconciliation in order to minimize DRPs. Furthermore, integrated maneuvers of hospital and industrial pharmacists can assist to solve the issues related to drug shortage and supply. A vigilant contact of industrial pharmacists with hospital administration will be of paramount importance to ensure the adequate and continuous supply of medications. During the current health crisis, drug and health regulatory authorities hold greater responsibilities as the pandemic is accompanied by various malpractices on drug use and disease management. Drug regulatory pharmacists should ensure adequate production of drugs in the industry, good selling practices, rational use of drugs, and quality and standards of medication supply and use. These activities can be effectively accomplished through intra- and interprofessional collaboration of drug regulatory pharmacists with other pharmacists and healthcare professionals. The integration of pharmacists into core healthcare teams during the current pandemic would facilitate positive patient outcomes, better team decision-making around drug therapy, improved continuity of care, and improved patient safety.

## Conclusive Remarks

During the current crisis, innovative and adaptive methods of practicing will be required across all health professions. The roles and activities underscored in the current manuscript are not exhaustive but serve to illustrate a range of areas in which pharmacists at different levels could make substantial contributions. These are currently implemented to varying extents across different countries. The activities of pharmacists during the COVID-19 pandemic widely differ from those have been described in previous pandemics and outbreaks during which pharmacists were primarily engaged in vaccination and disease education. This pandemic put more responsibilities on pharmacists due to disease novelty, its rapid transmission, associated morbidity and mortality, massive infodemic, misleading narratives, lack of vaccine or specific drugs, lack of treatment guidelines, and overwhelmed healthcare system.

Pharmacists can give full play to their professional expertise; analyze the current situation rationally; test, treat, and immunize; formulate telehealth policies expeditiously; and guarantee medication safety and rational use of drugs (43, 85, 88). In restructuring existing health services to respond to the current public health crisis, it is important that governments, public health bodies, and policy makers review existing services and make full use of any unrealized potential among pharmacists working in various sectors. In short, pharmacists could readily play a role in ramping up COVID-19 testing and treatment and, eventually, when available, providing the vaccine. Relaxing state phlebotomy laws could yield additional benefits, as drawing blood may be necessary in efforts to search for antibodies for COVID-19. Any restrictions on the ability of pharmacists to immunize using FDA-approved vaccines should also be reconsidered.

Since the impact of traditional and extended pharmacy services is not evaluated during the pandemic, well-structured and controlled studies are needed in this regard. Moreover, the extent of preparedness among pharmacists for any future outbreak is required to be ascertained. This review also identifies the poor coordination and collaboration of pharmacists working in different sectors with other frontline healthcare professionals dealing with COVID-19. These shortcomings should be considered while designing future research and implementing health policies for infectious diseases.

## Author Contributions

TM, YK, and NA: conception or design of the work. AAl, AL, and AAb: analysis or interpretation of studies for the work. TM, AL, AAb, and NT: drafted the work. NA, YK, AAl, and NT: revised the manuscript critically for important intellectual content. TM, YK, AL, and AAb: provided approval for publication of the content. All authors contributed to the article and approved the submitted version.

## Conflict of Interest

The authors declare that the research was conducted in the absence of any commercial or financial relationships that could be construed as a potential conflict of interest.
